# Dietary intake of antioxidant vitamins and risk of pancreatic cancer: the Japan public health center-based prospective study

**DOI:** 10.1007/s00394-025-03874-9

**Published:** 2026-02-14

**Authors:** Sayo Uesugi, Kumiko Kito, Taiki Yamaji, Motoki Iwasaki, Manami Inoue, Shoichiro Tsugane, Norie Sawada

**Affiliations:** 1 Nutrition Education, Department of Food Science, Otsuma Women’s University, Tokyo, Japan; 2 Division of Cohort Research, National Cancer Center Institute for Cancer Control, 5-1-1 Tsukiji, Chuo-Ku, Tokyo, 104-0045 Japan; 3 Division of Epidemiology, National Cancer Center Institute for Cancer Control, Tokyo, Japan; 4 Division of Prevention, National Cancer Center Institute for Cancer Control, Tokyo, Japan; 5 International University of Health and Welfare Graduate School of Public Health, Tokyo, Japan

**Keywords:** Pancreatic cancer, Antioxidant vitamins, Prospective study, Japanese, Body mass index, Dietary intake

## Abstract

**Purpose:**

Oxidative stress has been implicated in the development of pancreatic cancer, but the association between antioxidant vitamin intake and risk of pancreatic cancer in Asian populations has not been establish.

**Methods:**

We investigated the association between antioxidant vitamin intake and pancreatic cancer risk in a Japanese population based on the Japan Public Health Center-based Prospective Study, a cohort study including 89,693 Japanese men and women aged 45–74 years. Baseline data on medical history, lifestyle factors, and antioxidant vitamin intake were collected via validated questionnaires. Multivariable-adjusted hazard ratios (HRs) and 95% confidence intervals (CIs) were calculated for quartiles of antioxidant vitamin intake.

**Results:**

During an average follow-up was 15.1 years, we documented 581 incident pancreatic cancers. Our results did not indicate a potential inverse association between antioxidant vitamin intake and risk of pancreatic cancer. When stratified by body mass index, the inverse association between dietary retinol, β-carotene, α-carotene, and β-cryptoxanthin intake and risk of pancreatic cancer indicated a statistically significant association among those with BMI ≥ 25 but not BMI < 25. Corresponding multivariable hazard ratios for the highest versus lowest quartiles among BMI ≥ 25 were intake of retinol activity equivalents of 0.52 (0.31–0.86; *P* = 0.01), β-carotene equivalent of 0.53 (0.31–0.91; *P* = 0.01), α-carotene of 0.57 (0.33–0.97; *P* = 0.05), and β-cryptoxanthin of 0.56 (0.33–0.95; *P* = 0.02).

**Conclusion:**

Our findings suggests that intake of antioxidant vitamins, particularly retinol activity equivalents and β-carotene equivalents, may play a role in the prevention of pancreatic cancer in overweight subjects.

**Supplementary Information:**

The online version contains supplementary material available at 10.1007/s00394-025-03874-9.

## Background

Pancreatic cancer, a highly aggressive malignancy with a poor prognosis, is known for its delayed diagnosis and limited treatment options. An estimated 510,992 cases of pancreatic cancer were newly diagnosed worldwide in 2022 together with an estimated 467,409 deaths, ranking pancreatic cancer 6th among all malignant tumors. Asia accounted for the majority of both newly diagnosed cases (45.5%) and cancer-related deaths (45.4%) [1]. Moreover, the incidence of pancreatic cancer has steadily increased in recent years [2].

Interest in the potential role of oxidative stress and antioxidant systems in cancer development continues to grow. The ability of antioxidant vitamins, such as retinol, vitamin C, vitamin E, and β-carotene, to neutralize reactive oxygen species and protect against cellular damage has been extensively studied both in vitro and in vivo. Vitamin A prevents the DNA damage caused by reactive oxygen species (ROS) and which leads to carcinogenesis [3]. The primary influence of antioxidant vitamins lies in their modulation of ROS production, a factor critical to tumor-promoting inflammation [4]. These vitamins are commonly present in fruits, vegetables, and dietary supplements, and their consumption has been associated with various health benefits [5].

Several cohort studies conducted in Europe and North America have investigated the relationship between antioxidant vitamins and risk of developing pancreatic cancer [6–9]. To date, however, prospective findings have shown limited evidence of a preventive effect. Some case–control studies have suggested a potential protective effect [10–14], while other studies have shown no preventive effective [15–19]. Thus, findings to date are inconsistent and inconclusive. Additionally, despite differences in diet and physique between Asians and other populations, some case–control studies in Asian populations have indicated that intake of carotenoids, vitamin E, and vitamin C may have preventive effects [20, 21], but this question has not been prospectively studied in an Asian population.

Here, we aimed to identify the association between the intake of antioxidant vitamins and risk of pancreatic cancer among a Japan population.

## Methods

### Study population

**Fig. 1:**
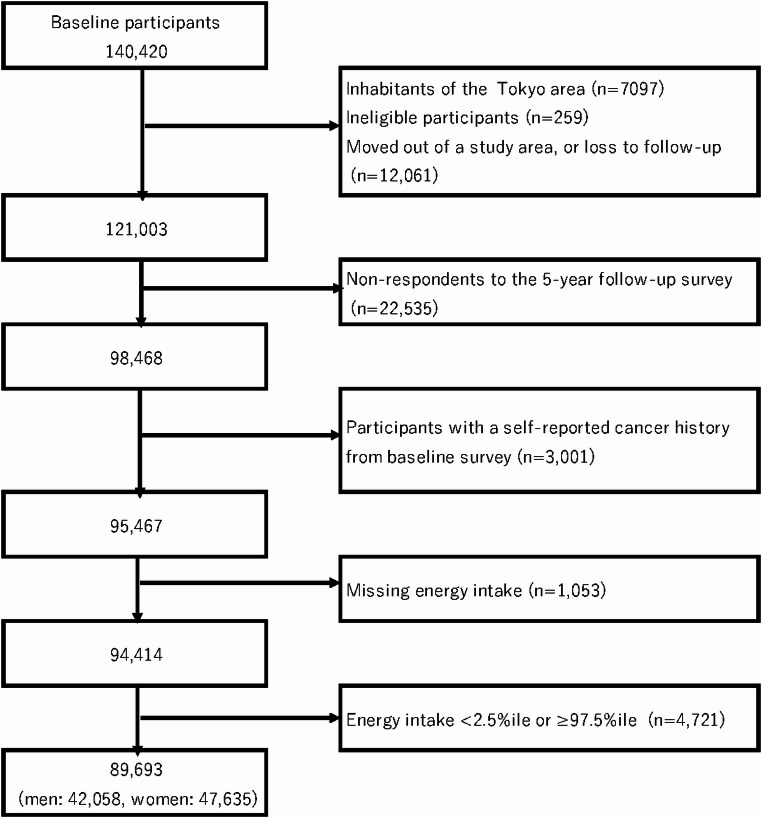
Flow chart of study participants

The Japan Public Health Center-based Prospective Study was initiated in 1990 (Cohort I) and 1993 (Cohort II). The study design has been described in detail elsewhere [22]. The study protocol was approved by the institutional review boards of the National Cancer Center, Tokyo. The study population was defined as all registered Japanese residents in 11 public health center areas, aged 40–59 years in Cohort I and 40–69 years in Cohort II. Residents were identified from population registries maintained by local municipalities. The cohort participants were surveyed three times by self-administered questionnaire. Because the 5-year follow-up survey had more comprehensive information on food intake frequency than the first survey, we defined it as the starting point for assessment of dietary exposure in the present analysis. The questionnaire also included items on lifestyle factors (smoking, alcohol drinking, and physical activity, etc.) and medical history, etc. We excluded 19,417 participants from the 5-year follow-up survey due to the lack of cancer incidence data in a single PHC area (n = 7,097); non-Japanese nationality (n = 52); late report of emigration before the 5-year follow-up survey (n = 188); incorrect date of birth (n = 7); duplicate enrollment (n = 12); and death or moving out of the PHC area before the 5-year follow-up survey (n = 12,061). Finally, we targeted a population of 121,003 participants. Of these, 98,468 men and women responded to the questionnaire (response rate 81.4%) and were included in the present study. Participants with a history of all cancer, including non-melanoma skin cancer, were excluded from the analysis (n = 3,001). Furthermore, we excluded 1,053 participants who did not respond to the dietary survey and 4,721 participants whose reported total energy intake was in the lowest 2.5% or highest 97.5% of the distribution. Consequently, 89,693 participants (42,058 men and 47,635 women) remained for the final analysis. (Fig. 1).

### Exposure assessment and variables

The food frequency questionnaire (FFQ) asked about the usual consumption of 138 foods and beverages during the previous year with standard portion/unit sizes and nine frequency categories [23].

Standard portion sizes were specified for each food item in the three amount choices of small (50 percent smaller), medium (same as the standard) and large (50 percent larger). Frequency responses were possible for each food item in the nine choices of ‘rarely’, ‘1–3 days/week’, ‘1–2 days/week’, ‘3–4 days/week’, and ‘5–6 days /week’, ‘once/day’, ‘2–3 times/day’, ‘4–6 times /day’, and ‘7 or more times/day’. The amount of each food consumed (grams/day) was then calculated from these responses. Energy and nutrient intakes, including retinol activity equivalents (μg), β-carotene equivalents (μg), α-carotene (μg), β-cryptoxanthin (μg), lycopene (μg), α-tocopherol (mg), and vitamin C (mg), for individuals were calculated using the Standardized Tables of Food Composition, 7th revised edition [24]. We selected retinol, β-carotene, α-carotene, β-cryptoxanthin, lycopene, α-tocopherol (vitamin E), and vitamin C as antioxidant vitamins for analysis. These nutrients are well recognized for their capacity to neutralize reactive oxygen species and thereby mitigate oxidative DNA damage, which is biologically relevant to carcinogenesis. Carotenoids such as β-carotene, α-carotene, β-cryptoxanthin, and lycopene, as well as α-tocopherol and vitamin C, have strong free radical–scavenging activities, while retinol, a preformed vitamin A, also contributes to antioxidant defense through its role in maintaining membrane stability and modulating cell differentiation. With the exception of lycopene, these nutrients are included in the Japanese Standard Tables of Food Composition (2020 edition) and could be estimated from our validated food frequency questionnaire (FFQ). Lycopene, although not listed in the standard tables, was estimated using an extended carotenoid database developed for the JPHC study. Evaluation of the validity of FFQ-based lycopene intake against dietary records and serum levels showed modest but significant correlations [25]. Previous JPHC studies have provided epidemiological evidence of the association of these antioxidant vitamins with lung, prostate, and liver cancer risk [26–28]. Based on this background, we included these vitamins in the present analysis of pancreatic cancer risk.

In 2015, the Ministry of Education, Culture, Sports, Science and Technology in Japan introduced a new conversion factor for β-carotene, replacing the former equivalent of retinol activity equivalents. Accordingly, the current estimation uses the following conversion factor:

$$ \begin{aligned} & {\mathrm{retinol}}\;{\mathrm{activity}}\;{\mathrm{equivalents}}\,{\mathrm{(}}\mu {\text{g RAE)}}\;{\text{ = }}\;{\mathrm{retinol}}\,{\mathrm{(}}\mu {\mathrm{g}})\;{\text{ + }}\;{\mathrm{1/12}}\;\beta {\text{ - crotene}}\,(\mu {\mathrm{g}}) \\ & \quad {\text{ + }}\;{\mathrm{1/24}}\;\alpha {\text{ - carotene}}\,{\mathrm{(}}\mu {\mathrm{g}})\;{\text{ + }}\;{\mathrm{1/24}}\;\beta {\text{ - cryptoxanthin}}\,(\mu {\mathrm{g}}) \\ \end{aligned} $$

The FFQ included questions on supplement use, but nutrient intake from supplements was not included in analysis because no comprehensive database for supplements was available.

Validation for nutrition data in the FFQ was evaluated using 14-day or 28-day dietary records, and reproducibility was evaluated using the 5-year follow-up questionnaire twice at an interval of approximately 1 year. Respective Spearman correlation coefficients (energy-adjusted) for validity in men and women were as follows: 0.47 and 0.31 for cohort I and 0.43 and 0.44 for cohort II for retinol equivalent; 0.43 and 0.31 for cohort I and 0.47 and 0.44 for cohort II for β-carotene equivalent; 0.29 and 0.52 for cohort I and 0.50 and 0.52 for cohort II for α-carotene; 0.43 and 0.29 for cohort I and 0.48 and 0.31 for cohort II for cryptoxanthin; 0.29 and 0.37 for cohort II for lycopene; 0.37 and 0.50 for cohort I and 0.24 and 0.37 for cohort II for α-tocopherol; 0.38 and 0.29 for cohort I and 0.46 and 0.44 for cohort II for vitamin C. For reproducibility, the coefficients were moderate: 0.83 and 0.43 for cohort I and 0.53 and 0.52 for cohort II for retinol equivalent; 0.44 and 0.44 for cohort I and 0.56 and 0.55 for cohort II for β-carotene equivalent; 0.48 and 0.42 for cohort I and 0.46 and 0.49 for cohort II for α-carotene; 0.49 and 0.58 for cohort I and 0.52 and 0.44 for cohort II for cryptoxanthin; 0.46 and 0.57 for cohort II for lycopene; 0.65 and 0.49 for cohort I and 0.58 and 0.35 for cohort II for α-tocopherol; and 0.67 and 0.49 for cohort I and 0.58 and 0.48 for cohort II for vitamin C [23, 29–31].

### Identification of pancreatic cancer

Cases of pancreatic cancer were determined through active patient information from major local hospitals in each study area and data linkage with population-based cancer registries. Death certificates were used as sources of additional information. We defined pancreatic cancer cases based on the International Classification of Diseases for Oncology, Third Edition codes C25.0–C25.9. Only confirmed primary malignant neoplasms of the pancreas were included, and secondary or metastatic pancreatic cancers were excluded.

### Statistical analysis

Person-years of follow-up were calculated for each subject from the starting point to the date of diagnosis, date of emigration from the study area, date of death, or end of the follow-up period (December 31, 2012 for Suita district and December 31, 2013 for all other districts), whichever occurred first. During a mean follow-up of 15.1 ± 4.3 years, 581 pancreatic cancer cases were identified. Hazard ratios (HRs) and 95 percent confidence intervals (CIs) were calculated for the categories of antioxidant vitamin (retinol activity equivalents, β-carotene equivalents, α-carotene, β-cryptoxanthin, lycopene, α-tocopherol, and vitamin C) intake and were adjusted for total energy intake using Cox proportional hazards models based on the residual method in quartiles, with the lowest consumption category as reference. Evaluation using Schoenfeld residuals showed that the proportional hazards assumption was violated for sex. Therefore, all Cox models were stratified by sex and study area (public health center). We conducted the initial analyses by adjusting for study area (10 public health center areas), age (continuous), and sex. In the multivariate model, we further adjusted for smoking status (never, ever, current < 20, 20–39, or ≥ 40 cigarettes/day, or missing), alcohol consumption (< 150 g, ≥ 150 g of ethanol/week or missing), body mass index (< 25.0, ≥ 25.0 kg/m^2^ or missing), family history of pancreatic cancer (yes or no), medical history of diabetes mellitus (yes or no), coffee consumption (< 1 cup/week, ≥ 1 cup/week), physical activity in metabolic equivalent task-hours/day (continuous), supplement use of multivitamins (no or yes), and energy-adjusted intakes of fish and processed meat (continuous).

In the multivariable models, we adjusted for potential confounders that have been reported in previous prospective cohort and case–control studies as risk factors for pancreatic cancer, including smoking status, alcohol intake, BMI, and history of diabetes. Smoking status was categorized into never, ever, and current smokers (< 20, 20–39, or ≥ 40 cigarettes/day, or missing) [32, 33]. Although cumulative exposure such as pack-years was not available in our baseline survey, this categorization allowed us to account for smoking intensity. BMI was included as a categorical variable (< 18.5, 18.5–24.9, 25.0–29.9, and ≥ 30 kg/m^2^). Because only 2,417 participants (2.6% of the cohort) had BMI ≥ 30, overweight and obesity were combined to ensure stable estimates. Stratified analyses were performed by sex-specific quartiles, smoking status (current and non-smokers), ethanol intake (unknown, 0, < 150 g/wk, ≥ 150 g/wk) and BMI (< 25.0 or ≥ 25.0 kg/m^2^) on the basis that antioxidant vitamins are consumed by obesity and smoking-related oxidative stress [1].

All statistical testing was conducted using SAS (SAS Institute, Inc., Cary, North Carolina). All p values were two-sided, and statistical significance was determined at the p < 0.05 level.

## Results

We confirmed 581 (323 men and 258 women) incident cases of pancreatic cancer during 1,357,043 person-years of follow-up (15.1 ± 4.3 years).

Median values and proportions of baseline characteristics are presented by sex in Table 1. Women had higher median intakes of all antioxidant vitamins than men. Men had higher BMI and were more likely to have diabetes, to smoke, and to consume larger amounts of alcohol, coffee, and total energy. As shown in Table 2, dietary intake of antioxidant vitamins was not associated with sex- and age-adjusted risk of pancreatic cancer, with these trends being not significant. Multivariable-adjusted HRs for highest versus lowest dietary antioxidant vitamin intake were 0.90 (95% CI, 0.70–1.14; p for trend = 0.35) for retinol activity equivalents; 1.02 (0.79–1.30; 0.86) for β-carotene equivalent; 1.11 (0.87–1.42; 0.58) for α-carotene; 0.89 (0.70–1.15; 0.25) for β-cryptoxanthin; 1.08 (0.85–1.37; 0.32) for lycopene; 1.02 (0.78–1.35; 0.72) for α-tocopherol; and 1.10 (0.84–1.43; 0.98) for vitamin C. Further, no significant associations between dietary antioxidant vitamin intake and pancreatic cancer risk were observed in either men or women after participants were stratified by sex (Table 3).

**Table 1:** Baseline characteristics of participants and intake of antioxidant vitamins

	Men	Women	*P* value^1^
n	42,058	47,635	
*Vitamin intake (IQR)*			
Retinol Eq (μg/day)	711 (460–1039)	745 (523–1044)	< 0.01
β carotene Eq. (μg/day)	3065 (1919–4676)	4181 (2883–5900)	< 0.01
α carotene. (μg/day)	397 (191–753)	567 (307–962)	< 0.01
β cryptoxanthin. (μg/day)	678 (308–1238)	1027 (552–1791)	< 0.01
Lycopene (μg/day)	899 (295–3417)	929 (390–2766)	< 0.01
α tocoqherol. (mg/day)	6.4 (5.1–7.9)	7 (5.9–8.3)	< 0.01
Vitamin C. (mg/day)	103 (70–147)	135 (98–183)	< 0.01
*characteristics*			
Age (years), median (IQR)	51 (45–57)	51 (45–57)	< 0.01
BMI (kg/m^2^), median (IQR)	23.4 (21.6–25.4)	23.2 (21.3–25.4)	< 0.01
History of diabetes mellitus, %	9.4	4.5	< 0.01
Family history of pancreatic cancer, %	0.3	0.4	0.04
Current smoker, %	46.4	5.5	< 0.01
Ethanol intake ≥ 150 g/wk, %	48.9	2.8	< 0.01
Coffee intake > 1 cup/wk, %	75.4	71.4	< 0.01
Physical activity, MET-h/d, median (IQR)	31.3 (24.3–35.5)	31.3 (24.7–31.9)	< 0.01
*Dietary intake, median (IQR)*			
Total energy (kcal/d)	2092 (1702–2561)	1786 (1460–2195)	< 0.01
Processed meat (g/d)	3.8 (1.4–8.4)	3.9 (1.5–8.1)	0.17
Fish (g/d)	77.8 (52.2–111.6)	75.5 (51.6–105.3)	< 0.01

**Table 2:** Hazard ratios for pancreatic cancer incidence by quartile of intake of antioxidant vitamins

	Lowest	Second	Third	Highest	p trend
*Retinol Eq*					
Median intake (IQR), μg/day	364 (279–430)	607 (549–666)	865 (793–945)	1337 (1164–1666)	
Cases	157	148	138	138	
Person Years	3,37,691	3,39,918	3,41,434	3,38,000	
Model 1 HR (95%CI)	Ref	0.96 (0.76–1.20)	0.90 (0.71–1.13)	0.88 (0.70–1.11)	0.24
Model 2 HR (95%CI)	Ref	0.93 (0.74–1.17)	0.89 (0.70–1.13)	0.90 (0.70–1.14)	0.26
*β carotene Eq*					
Median intake (IQR), μg/day	1642 (1186–2009)	2995 (2668–3326)	4419 (4023–4869)	7018 (6070–8778)	
Cases	145	147	135	154	
Person Years	3,34,712	3,39,826	3,42,192	3,40,314	
Model 1 HR (95%CI)	Ref	1.00 (0.79–1.26)	0.90 (0.71–1.15)	1.01 (0.80–1.28)	0.86
Model 2 HR (95%CI)	Ref	1.02 (0.81–1.30)	0.90 (0.70–1.15)	1.02 (0.79–1.30)	0.86
*α carotene*					
Median intake (IQR), μg/day	128 (75–183)	351 (295–414)	649 (561–751)	1270 (1033–1735)	
Cases	145	150	136	150	
Person Years	3,34,529	3,39,918	3,42,057	3,40,539	
Model 1 HR (95%CI)	Ref	1.08 (0.86–1.36)	0.98 (0.77–1.24)	1.07 (0.84–1.35)	0.79
Model 2 HR (95%CI)	Ref	1.09 (0.86–1.39)	1.00 (0.78–1.28)	1.11 (0.87–1.42)	0.58
*β cryptoxanthin*					
Median intake (IQR), μg/day	197 (102–306)	624 (519–735)	1134 (984–1313)	2410 (1872–3448)	
Cases	156	144	130	151	
Person Years	3,37,180	3,43,451	3,42,819	3,33,593	
Model 1 HR (95%CI)	Ref	0.90 (0.72–1.13)	0.78 (0.62–0.99)	0.89 (0.70–1.12)	0.20
Model 2 HR (95%CI)	Ref	0.91 (0.72–1.15)	0.80 (0.62–1.02)	0.89 (0.70–1.15)	0.25
*Lycopene*					
Median intake (IQR), μg/day	157 (72–239)	549 (430–699)	1589 (1175–2209)	5502 (4061–8186)	
Cases	149	128	151	153	
Person Years	3,39,688	3,39,380	3,41,517	3,36,458	
Model 1 HR (95%CI)	Ref	0.92 (0.72–1.16)	1.04 (0.83–1.31)	1.08 (0.86–1.35)	0.35
Model 2 HR (95%CI)	Ref	0.93 (0.73–1.19)	1.08 (0.85–1.37)	1.08 (0.85–1.37)	0.32
*α tocopherol*					
Median intake (IQR), mg/day	4.6 (4.0–5.1)	6.1 (5.8–6.4)	7.3 (7.0–7.7)	9.2 (8.6–10.4)	
Cases	141	143	156	141	
Person Years	3,38,913	3,41,406	3,40,430	3,36,294	
Model 1 HR (95%CI)	Ref	1.05 (0.83–1.32)	1.14 (0.90–1.43)	0.99 (0.78–1.25)	0.91
Model 2 HR (95%CI)	Ref	1.11 (0.86–1.42)	1.24 (0.96–1.60)	1.02 (0.78–1.35)	0.72
*Vitamin C*					
Median intake (IQR), mg/day	62 (47–73)	101 (92–110)	141 (130–153)	209 (185–246)	
Cases	127	160	138	156	
Person Years	3,36,544	3,40,775	3,41,617	3,38,107	
Model 1 HR (95%CI)	Ref	1.19 (0.94–1.50)	0.99 (0.77–1.26)	1.07 (0.84–1.37)	0.98
Model 2 HR (95%CI)	Ref	1.26 (0.98–1.61)	1.01 (0.78–1.31)	1.10 (0.84–1.43)	0.98

Model 1 was stratified by sex and study area and adjusted for age

Model 2 was further adjusted for BMI (< 25, ≥ 25), smoking status (Never, Ever, Current: < 20, 20–40, ≥ 40), history of diabetes mellitus (“yes” or “no”), family history of pancreatic cancer (“yes” or “no”), ethanol intake (unknown, 0, < 150 g/wk, ≥ 150 g/wk), coffee intake (< 1 cup/week, ≥ 1 cup/week), METs, processed meat intake, and fish intake

**Table 3:** Hazard ratios (HR) and 95% confidence interval (CI) of pancreatic cancer according to quintile of dietary vitamin intakes by sex

	Men			n = 42,058	
	Q1	Q2	Q3	Q4^1^	p trend
*Retinol Eq*					
Median intake (IQR), μg/day	345 (256–418)	607 (549–665)	866 (795–946)	1356 (1169–1695)	
Cases	78	85	79	81	
Person Years	1,55,012	1,54,930	1,55,659	1,52,434	
Model 1 HR (95%CI)	Ref	1.05 (0.77–1.43)	0.98 (0.72–1.35)	1.01 (0.73–1.38)	0.93
Model 2 HR (95%CI)	Ref	1.01 (0.74–1.39)	0.93 (0.67–1.30)	1.01 (0.73–1.41)	0.94
*β carotene Eq*					
Median intake (IQR), μg/day	1568 (1112–1961)	2958 (2638–3297)	4383 (3994–4830)	6971 (6043–8796)	
Cases	69	85	71	98	
Person Years	1,54,191	1,55,689	1,55,220	1,52,935	
Model 1 HR (95%CI)	Ref	1.14 (0.83–1.56)	0.90 (0.64–1.25)	1.20 (0.87–1.64)	0.51
Model 2 HR (95%CI)	Ref	1.18 (0.85–1.65)	0.96 (0.68–1.36)	1.25 (0.89–1.75)	0.38
*α carotene*					
Median intake (IQR), μg/day	121 (72–178)	343 (290–409)	641 (556–742)	1257 (1028–1718)	
Cases	71	78	80	94	
Person Years	1,53,559	1,55,630	1,55,221	1,53,623	
Model 1 HR (95%CI)	Ref	1.08 (0.78–1.49)	1.08 (0.78–1.49)	1.24 (0.91–1.70)	0.20
Model 2 HR (95%CI)	Ref	1.10 (0.78–1.53)	1.14 (0.82–1.60)	1.33 (0.96–1.86)	0.09
*β cryptoxanthin*					
Median intake (IQR), μg/day	179 (92–295)	614 (513–726)	1120 (977–1298)	2311 (1829–3273)	
Cases	79	82	75	87	
Person Years	1,54,863	1,56,972	1,55,282	1,50,917	
Model 1 HR (95%CI)	Ref	0.98 (0.72–1.34)	0.86 (0.62–1.18)	0.95 (0.69–1.29)	0.56
Model 2 HR (95%CI)	Ref	0.97 (0.70–1.33)	0.87 (0.63–1.21)	0.97 (0.70–1.35)	0.72
*Lycopene*					
Median intake (IQR), μg/day	147 (64–230)	536 (423–683)	1657 (1199–2310)	5567 (4103–8272)	
Cases	73	80	79	91	
Person Years	1,55,424	1,53,580	1,55,412	1,53,618	
Model 1 HR (95%CI)	Ref	1.11 (0.81–1.52)	1.04 (0.76–1.44)	1.29 (0.95–1.76)	0.16
Model 2 HR (95%CI)	Ref	1.11 (0.80–1.54)	1.08 (0.78–1.50)	1.27 (0.92–1.75)	0.19
*α tocopherol*					
Median intake (IQR), mg/day	4.5 (3.8–5.0)	6.1 (5.8–6.4)	7.3 (7.0–7.7)	9.3 (8.6–10.5)	
Cases	69	90	76	88	
Person Years	1,56,064	1,56,004	1,54,359	1,51,607	
Model 1 HR (95%CI)	Ref	1.23 (0.90–1.69)	1.01 (0.73–1.40)	1.14 (0.82–1.57)	0.75
Model 2 HR (95%CI)	Ref	1.30 (0.93–1.82)	1.09 (0.76–1.56)	1.22 (0.83–1.77)	0.58
*Vitamin C*					
Median intake (IQR), mg/day	59 (45–71)	100 (91–110)	140 (130–152)	206 (184–243)	
Cases	57	91	81	94	
Person Years	1,54,605	1,55,630	1,55,362	1,52,437	
Model 1 HR (95%CI)	Ref	1.43 (1.03–2.00)	1.18 (0.84–1.66)	1.26 (0.90–1.77)	0.47
Model 2 HR (95%CI)	Ref	1.50 (1.06–2.12)	1.21 (0.85–1.74)	1.34 (0.93–1.92)	0.36

	Women			n = 47,635	
	Q1	Q2	Q3	Q4^1^	p trend
*Retinol Eq*					
Median intake (IQR), μg/day	383 (306–440)	606 (549–666)	864 (792–944)	1322 (1161–1642)	
Cases	73	72	56	57	
Person Years	1,84,080	1,83,867	1,85,563	1,85,498	
Model 1 HR (95%CI)	Ref	0.99 (0.72–1.38)	0.78 (0.55–1.10)	0.78 (0.55–1.11)	0.08
Model 2 HR (95%CI)	Ref	0.97 (0.69–1.36)	0.79 (0.55–1.14)	0.79 (0.55–1.14)	0.12
*β carotene Eq*					
Median intake (IQR), μg/day	1778 (1349–2080)	3028 (2699–3350)	4442 (4043–4894)	7036 (6086–8771)	
Cases	63	71	54	70	
Person Years	1,83,082	1,85,323	1,85,649	1,84,955	
Model 1 HR (95%CI)	Ref	1.09 (0.77–1.53)	0.79 (0.55–1.14)	0.97 (0.69–1.37)	0.48
Model 2 HR (95%CI)	Ref	1.08 (0.76–1.54)	0.75 (0.51–1.10)	0.89 (0.62–1.28)	0.24
*α carotene*					
Median intake (IQR), μg/day	136 (80–188)	357 (300–417)	655 (564–754)	1277 (1036–1744)	
Cases	77	55	60	66	
Person Years	1,82,584	1,85,427	1,85,693	1,85,305	
Model 1 HR (95%CI)	Ref	0.74 (0.52–1.04)	0.80 (0.57–1.12)	0.85 (0.61–1.19)	0.41
Model 2 HR (95%CI)	Ref	0.70 (0.49–1.01)	0.77 (0.54–1.10)	0.82 (0.58–1.17)	0.34
*β cryptoxanthin*					
Median intake (IQR), μg/day	221 (123–321)	635 (526–743)	1143 (992–1325)	2464 (1893–3529)	
Cases	66	67	58	67	
Person Years	1,85,141	1,87,732	1,85,548	1,80,588	
Model 1 HR (95%CI)	Ref	0.94 (0.67–1.32)	0.76 (0.53–1.09)	0.82 (0.58–1.17)	0.17
Model 2 HR (95%CI)	Ref	0.93 (0.65–1.32)	0.76 (0.52–1.09)	0.76 (0.52–1.10)	0.09
*Lycopene*					
Median intake (IQR), μg/day	167 (81–249)	560 (438–710)	1543 (1161–2140)	5439 (4004–8104)	
Cases	67	59	64	68	
Person Years	1,85,677	1,84,495	1,85,441	1,83,395	
Model 1 HR (95%CI)	Ref	0.94 (0.66–1.34)	0.99 (0.70–1.40)	1.08 (0.76–1.51)	0.63
Model 2 HR (95%CI)	Ref	0.98 (0.68–1.41)	0.99 (0.69–1.43)	1.11 (0.78–1.58)	0.57
*α tocopherol*					
Median intake (IQR), mg/day	4.8 (4.3–5.2)	6.1 (5.8–6.4)	7.4 (7.0–7.7)	9.2 (8.6–10.3)	
Cases	65	59	75	59	
Person Years	1,85,335	1,85,638	1,84,443	1,83,592	
Model 1 HR (95%CI)	Ref	0.93 (0.65–1.33)	1.17 (0.84–1.64)	0.89 (0.62–1.27)	0.87
Model 2 HR (95%CI)	Ref	0.98 (0.67–1.41)	1.23 (0.86–1.77)	0.85 (0.57–1.27)	0.72
*Vitamin C*					
Median intake (IQR), mg/day	65 (53–75)	102 (93–111)	141 (131–154)	210 (186–248)	
Cases	57	66	68	67	
Person Years	1,85,702	1,85,244	1,84,854	1,83,209	
Model 1 HR (95%CI)	Ref	1.07 (0.75–1.54)	1.04 (0.73–1.49)	0.96 (0.67–1.39)	0.77
Model 2 HR (95%CI)	Ref	1.12 (0.78–1.63)	1.05 (0.72–1.52)	0.90 (0.62–1.33)	0.49

^1^Sex-specific quartiles

Model 1 was stratified by study area and adjusted for age

Model 2 was further adjusted for BMI (< 25, ≥ 25), smoking status (Never, Ever, Current: < 20, 20–40, ≥ 40), history of diabetes mellitus (“yes” or “no”), family history of pancreatic cancer (“yes” or “no”), ethanol intake (unknown, 0, < 150 g/wk, ≥ 150 g/wk), coffee intake (< 1 cup/week, ≥ 1 cup/week), METs, processed meat intake, and fish intake

In analyses stratified by BMI (Table 4), we observed a statistically significant inverse association between the intake of some antioxidant vitamins and risk of pancreatic cancer in participants with BMI ≥ 25 kg/m^2^. HRs of the highest compared with the lowest intake were 0.52 (95% CI, 0.31–0.86; P for trend = 0.01) for retinol activity equivalents; 0.53 (0.31–0.91; 0.01) for β-carotene equivalent; 0.57 (0.33–0.97; 0.05) for α-carotene; and 0.56 (0.33–0.95; 0.02) for β-cryptoxanthin. In contrast, we did not identify an inverse association in subjects with < 25 kg/m^2^. Interactions between intake of retinol equivalent, β-carotene equivalent α-carotene and β-cryptoxanthin, and BMI were statistically significant (p-interaction: 0.06, 0.02, 0.05, 0.12).

**Table 4:** Hazard ratios (HR) and 95% confidence interval (CI) of pancreatic cancer according to quintile of dietary vitamin intakes by BMI

	BMI < 25.0			n = 62,658		
	Q1	Q2	Q3	Q4	p trend	p interaction^1^
*Retinol Eq*						
Median intake (IQR), μg/day	367 (283–431)	607 (550–666)	864 (792–944)	1337 (1163–1666)		
Cases	99	109	101	108		
Person Years	2,33,990	2,40,361	2,38,502	2,32,196		
Model 1 HR (95%CI)	Ref	1.11 (0.84–1.46)	1.04 (0.79–1.38)	1.10 (0.83–1.45)	0.62	
Model 2 HR (95%CI)	Ref	1.05 (0.79–1.39)	1.01 (0.76–1.34)	1.07 (0.80–1.42)	0.36	0.06
*β carotene Eq*						
Median intake (IQR), μg/day	1647 (1193–2010)	2998 (2671–3325)	4417 (4024–4867)	6995 (6066–8745)		
Cases	92	106	98	121		
Person Years	2,31,868	2,39,010	2,39,922	2,34,249		
Model 1 HR (95%CI)	Ref	1.15 (0.87–1.52)	1.06 (0.79–1.42)	1.30 (0.98–1.72)	0.11	
Model 2 HR (95%CI)	Ref	1.12 (0.84–1.50)	1.03 (0.76–1.39)	1.26 (0.94–1.69)	0.20	0.02
*α carotene*						
Median intake (IQR), μg/day	129 (77–183)	352 (295–413)	649 (561–750)	1265 (1032–1721)		
Cases	90	110	96	121		
Person Years	2,32,817	2,40,063	2,39,199	2,32,970		
Model 1 HR (95%CI)	Ref	1.25 (0.95–1.66)	1.11 (0.83–1.49)	1.40 (1.05–1.85)	0.05	
Model 2 HR (95%CI)	Ref	1.25 (0.94–1.66)	1.10 (0.82–1.49)	1.39 (1.04–1.87)	0.06	0.05
*β cryptoxanthin*						
Median intake (IQR), μg/day	198 (103–307)	626 (521–736)	1132 (983–1311)	2399 (1865–3418)		
Cases	107	103	93	114		
Person Years	2,32,335	2,39,850	2,41,980	2,30,883		
Model 1 HR (95%CI)	Ref	0.95 (0.72–1.24)	0.83 (0.63–1.10)	1.05 (0.79–1.38)	0.97	
Model 2 HR (95%CI)	Ref	0.91 (0.69–1.21)	0.82 (0.61–1.10)	1.04 (0.78–1.38)	0.98	0.12
*Lycopene*						
Median intake (IQR), μg/day	159 (74–241)	551 (430–703)	1579 (1171–2207)	5500 (4055–8203)		
Cases	103	90	115	109		
Person Years	2,29,239	2,40,585	2,42,943	2,32,282		
Model 1 HR (95%CI)	Ref	0.91 (0.68–1.21)	1.12 (0.86–1.47)	1.10 (0.84–1.44)	0.27	
Model 2 HR (95%CI)	Ref	0.89 (0.66–1.19)	1.14 (0.86–1.50)	1.08 (0.82–1.43)	0.28	0.37
*α tocopherol*						
Median intake (IQR), mg/day	4.6 (4.0–5.1)	6.1 (5.8–6.4)	7.3 (7.0–7.7)	9.2 (8.6–10.3)		
Cases	92	104	116	105		
Person Years	2,37,330	2,38,466	2,40,590	2,28,662		
Model 1 HR (95%CI)	Ref	1.19 (0.90–1.59)	1.31 (1.00–1.73)	1.18 (0.88–1.57)	0.21	
Model 2 HR (95%CI)	Ref	1.24 (0.92–1.67)	1.40 (1.03–1.89)	1.18 (0.85–1.63)	0.28	0.19
*Vitamin C*						
Median intake (IQR), mg/day	62 (48–73)	101 (92–110)	141 (130–153)	208 (185–245)		
Cases	83	115	102	117		
Person Years	2,29,296	2,38,547	2,40,603	2,36,601		
Model 1 HR (95%CI)	Ref	1.30 (0.97–1.72)	1.13 (0.84–1.51)	1.27 (0.94–1.71)	0.27	
Model 2 HR (95%CI)	Ref	1.33 (0.99–1.79)	1.13 (0.83–1.54)	1.26 (0.93–1.73)	0.35	0.25

	BMI ≥ 25.0			n = 24,788		
	Q1	Q2	Q3	Q4	p trend	p interaction^1^
*Retinol Eq*						
Median intake (IQR), μg/day	361 (275–428)	605 (549–664)	866 (794–946)	1333 (1165–1654)		
Cases	49	35	32	24		
Person Years	94,173	92,875	96,714	98,240		
Model 1 HR (95%CI)	Ref	0.71 (0.46–1.10)	0.63 (0.40–0.98)	0.47 (0.29–0.76)	< 0.01	
Model 2 HR (95%CI)	Ref	0.69 (0.44–1.09)	0.66 (0.41–1.05)	0.52 (0.31–0.86)	0.01	0.06
*β carotene Eq*						
Median intake (IQR), μg/day	1648 (1185–2014)	2986 (2662–3328)	4423 (4019–4874)	7045 (6081–8833)		
Cases	42	40	33	25		
Person Years	93,627	94,174	95,614	98,587		
Model 1 HR (95%CI)	Ref	0.88 (0.57–1.36)	0.69 (0.43–1.10)	0.50 (0.30–0.84)	< 0.01	
Model 2 HR (95%CI)	Ref	0.94 (0.59–1.48)	0.72 (0.44–1.18)	0.53 (0.31–0.91)	0.01	0.02
*α carotene*						
Median intake (IQR), μg/day	129 (77–184)	349 (294–415)	651 (561–753)	1281 (1036–1756)		
Cases	44	37	35	24		
Person Years	91,078	93,945	96,669	1,00,310		
Model 1 HR (95%CI)	Ref	0.84 (0.54–1.31)	0.78 (0.50–1.22)	0.53 (0.32–0.88)	0.02	
Model 2 HR (95%CI)	Ref	0.91 (0.58–1.44)	0.86 (0.54–1.37)	0.57 (0.33–0.97)	0.05	0.05
*β cryptoxanthin*						
Median intake (IQR), μg/day	198 (104–308)	620 (515–732)	1139 (987–1315)	2414 (1876–3481)		
Cases	42	38	31	29		
Person Years	97,167	97,585	93,820	93,431		
Model 1 HR (95%CI)	Ref	0.84 (0.54–1.31)	0.65 (0.40–1.05)	0.56 (0.34–0.92)	0.01	
Model 2 HR (95%CI)	Ref	0.90 (0.57–1.42)	0.66 (0.40–1.09)	0.56 (0.33–0.95)	0.02	0.12
*Lycopene*						
Median intake (IQR), μg/day	157 (76–238)	544 (433–689)	1614 (1186–2227)	5451 (4051–7983)		
Cases	38	35	33	34		
Person Years	99,617	92,433	92,248	97,704		
Model 1 HR (95%CI)	Ref	1.00 (0.63–1.58)	0.89 (0.56–1.42)	0.88 (0.55–1.41)	0.52	
Model 2 HR (95%CI)	Ref	1.07 (0.66–1.73)	1.00 (0.61–1.62)	0.94 (0.58–1.53)	0.74	0.37
*α tocopherol*						
Median intake (IQR), mg/day	4.6 (4.0–5.1)	6.1 (5.8–6.4)	7.4 (7.0–7.7)	9.3 (8.6–10.5)		
Cases	41	33	39	27		
Person Years	92,538	96,036	93,472	99,957		
Model 1 HR (95%CI)	Ref	0.75 (0.47–1.19)	0.89 (0.57–1.40)	0.57 (0.35–0.94)	0.07	
Model 2 HR (95%CI)	Ref	0.82 (0.50–1.34)	0.99 (0.60–1.63)	0.61 (0.34–1.08)	0.18	0.19
*Vitamin C*						
Median intake (IQR), mg/day	62 (47–73)	101 (92–110)	140 (130–152)	209 (185–248)		
Cases	37	38	34	31		
Person Years	99,292	95,156	94,449	93,105		
Model 1 HR (95%CI)	Ref	0.97 (0.62–1.54)	0.81 (0.50–1.31)	0.69 (0.42–1.14)	0.11	
Model 2 HR (95%CI)	Ref	1.11 (0.69–1.78)	0.84 (0.50–1.40)	0.75 (0.44–1.28)	0.17	0.25

Model 1 was stratified by sex and study area and adjusted for age

Model 2 was further adjusted for smoking status (Never, Ever, Current: < 20, 20–40, ≥ 40), history of diabetes mellitus (“yes” or “no”), family history of pancreatic cancer (“yes” or “no”), ethanol intake (unknown, 0, < 150 g/wk, ≥ 150 g/wk), coffee intake (< 1 cup/week, ≥ 1 cup/week), METs, processed meat intake, and fish intake

^1^p for interaction by BMI category (< 25, ≥ 25)

In analyses stratified by smoking status and consumption of alcohol, the direction of the association did not differ among strata for each factor, and P values for trend were not statistically significant in these analyses (Supplementary Tables S2–S3). Furthermore, the results were unchanged after adjustment for vitamin supplement use (data not shown).

## Discussion

In this large population-based prospective study in Japanese men and women, we found no overall association between dietary intakes of retinol activity equivalents, β-carotene equivalents, α-carotene, β-cryptoxanthin, lycopene, α-tocopherol, and vitamin C and pancreatic cancer. However, inverse associations with retinol activity equivalents, β-carotene equivalents and β-cryptoxanthin with risk of pancreatic cancer were statistically significant among participants with a BMI of 25 kg/m^2^ or more.

Although a meta-analysis of 18 case–control and 4 cohort studies found that dietary vitamin A, carotene or β-carotene was inversely associated with pancreatic cancer risk [6], the prospective studies subgroup in that study showed no association. Furthermore, after stratification of all 22　included studies by ethnicity, the reduced risk of pancreatic cancer seen with increasing vitamin A intake was more pronounced among Caucasian than Asian populations [6]. Supporting this meta-analysis, our present cohort study also generally showed no association in a Japanese population.

A recent meta-analysis of 10 reports, including 4 prospective studies, showed that the intake of vitamin E, including supplements, was associated with reduced risk of pancreatic cancer [9]. However, three of the four prospective studies included in this meta-analysis showed that there was no association with risk of pancreatic cancer and intake from foods only [19, 34, 35]. Our finding of no association between intake of α-tocopherol and risk of pancreatic cancer is consistent with these previous studies.

A previous meta-analysis study showed that the summary relative risk for case–control studies (14 studies) and cohort studies (6 studies) on comparison of the highest versus lowest categories of vitamin C intake was 0.58 (95% CI: 0.52–0.66) and 0.93 (0.78–1.11), respectively [7]. Our cohort study also showed no association, supporting the results of this meta-analysis.

Our results showed no association between antioxidant nutrients and pancreatic cancer in total subjects, but did show an inverse association only among participants with a BMI of 25 kg/m^2^ or more. Although a meta-analysis demonstrated that dietary vitamin A intake was also inversely associated with the risk of pancreatic cancer [8], this meta-analysis included five studies in America and five in Europe, and had a higher BMI than that in our population. There is strong evidence that being overweight or obese increases risk of pancreatic cancer [36] and obese and overweight individuals have a chronic low-level inflammatory state in the body due to the release of inflammatory cytokines from fat cells [37, 38]. Anti-oxidant vitamins, such as retinol, carotene, and β-cryptoxanthin, which are fat-soluble, might be particularly effective in the overweight and obesity population under a chronic inflammatory state. The reason why our study showed no association in total subjects may partly be explained by the fact that the proportion of obesity (BMI ≥ 25) in Japanese is much lower than that in Western countries (for example, 24.0% in Japan, 72.4% in US [39]. Considering that vitamin C is water-soluble, it is not clear that vitamin C effects overweight subjects only, and further studies are needed.

The mechanism by which provitamin A, namely β-carotene, α-carotene, and β-cryptoxanthin, acts on pancreatic cancer as vitamin A is as follows. First, retinoids induce apoptosis in pancreatic cancer cells, and suppress pancreatic cancer growth via the activation of retinoic acid receptor-gamma, suggesting that vitamin A and its metabolites may play a protective role against pancreatic cancer [40]. Second, a metabolite of vitamin A may help shut down activated pancreatic stellate cells and prevent the formation of connective tissue around tumors, thereby exerting an anti-tumor effect [37, 41]. Furthermore, as shown in several preclinical studies, retinols, which are a kind of vitamin A, play a role in many types of signaling in pathways related to cell growth, adhesion and migration [41–43]. Indeed, retinoic acid was recently shown to inhibit pancreatic cancer cell migration and epithelial-mesenchymal transition by decreasing the expression of interleukin 6 (IL-6) in cancer-associated fibroblast (CAFs) cells [44].

The strengths of this study derive from the features of the JPHC Study, namely its prospective design with long follow-up; large general population of participants with a high response rate and high proportion of follow-up participants; and the availability of food intakes estimated using a detailed, validated FFQ. Several limitations also warrant mention. First, lifestyle factors, including food intake, were estimated from a 5-year follow-up survey conducted at a single time point only, meaning that we could not assess dietary change. Second, the number of incident cases of pancreatic cancer may not have been sufficient for analyses stratified by exposure subgroup and risk factor, and the stratified analysis findings may accordingly be in part due to chance. Third, our association findings may have been affected by residual confounding effects and unmeasured confounding variables. In addition, the validation study of our FFQ showed that the correlation coefficients for the validity of antioxidant vitamins ranged from 0.24 to 0.52, and reproducibility coefficients ranged from 0.24 to 0.83. Several nutrients had coefficients below 0.3. Such low coefficients may have influenced the observed associations through potential exposure misclassification. Furthermore, although we asked participants about the use of individual vitamin supplements as well as multivitamins, this information was not incorporated into the calculation of total vitamin intake. While additional adjustment for multivitamin use did not materially alter the results (data not shown), the lack of estimation of individual vitamin intake from supplements may have introduced exposure misclassification and potential bias.

In conclusion, our findings suggest that dietary intake of antioxidant vitamins is not significantly associated with the overall risk of pancreatic cancer in the general Japanese population. A possible inverse association was observed among individuals with a BMI of 25 kg/m^2^ or higher, suggesting that consumption of antioxidant vitamin-rich foods may help prevent pancreatic cancer in this subgroup. However, because obesity is itself a major risk factor for pancreatic cancer and many other chronic diseases, maintaining an appropriate body weight remains essential.

## Supplementary Information

Below is the link to the electronic supplementary material.

Supplementary file1 (PDF 148 KB)

## Data Availability

Information on how to access JPHC Study data is provided at https://epi.ncc.go.jp/en/jphc/index.html.

## Declarations

### Generative AI and AI-assisted technologies in the writing process

During the preparation of this work the authors did not use AI or AI-assisted technologies.
