# Supplementary material for: Dietary intake of antioxidant vitamins and risk of pancreatic cancer: the Japan public health center-based prospective study
Source: Eur J Nutr. 2026 Feb 14;65(2):57. doi: 10.1007/s00394-025-03874-9 (PMC12906578; doi:10.1007/s00394-025-03874-9)
Supplement: Supplementary file 1 — Supplementary file1 (PDF 148 KB) [file 394_2025_3874_MOESM1_ESM.pdf]

Supplementary Table. 1 Baseline characteristics of participants by intake of antioxidant vitamins

	Lowest	Second	Third	Highest	P value [1]
**Retinol Eq (μg/day)**	< 255.9	255.9-490.8	490.8-1042.3	≥ 1042.3	
Age (years), median (IQR)	51 (44-57)	51 (45-57)	51 (45-57)	52 (46-58)	<0.01
Men, %	53.6	43.6	43.8	46.6	<0.01
BMI (kg/m^2), median (IQR)	23.3 (21.4-25.4)	23.2 (21.4-25.3)	23.4 (21.5-25.4)	23.4 (21.5-25.4)	<0.01
History of diabetes mellitus, %	6.0	6.3	6.7	8.1	<0.01
Family history of pancreatic cancer, %	0.4	0.4	0.4	0.3	0.14
Current smoker, %	31.2	22.6	22.5	23.1	<0.01
Ethanol intake ≥150 g/wk, %	33.1	23.7	22.1	18.8	<0.01
Coffee intake >1 cup/wk, %	72.3	75.3	74.9	71.0	<0.01
Physical activity, MET-h/d, median (IQR)	31.3 (24.3-33.7)	31.3 (24.3-33.3)	31.3 (24.7-33.7)	31.3 (24.7-33.1)	0.01
Dietary intake, median (IQR)					
Total energy (kcal/d)	1894 (1495-2384)	1961 (1586-2427)	1948 (1589-2383)	1835 (1484-2274)	<0.01
Processed meat (g/d)	3.0 (1.0-7.4)	3.5 (1.3-8.6)	4.3 (1.7-9.1)	4.3 (2.0-9.6)	<0.01
Fish (g/d)	66.2 (39.3-107.6)	78.3 (48.3-122.6)	80.1 (50.1-122.4)	79.5 (49.4-123.2)	<0.01
**β carotene Eq (μg/day)**	< 1071.1	1071.1-2345.9	2345.9-5390.3	≥ 5390.3	
Age (years), median (IQR)	50 (44-56)	51 (45-57)	52 (46-58)	53 (47-59)	<0.01
Men, %	65.6	48.6	38.9	34.5	<0.01
BMI (kg/m^2), median (IQR)	23.3 (21.4-25.4)	23.3 (21.5-25.3)	23.3 (21.5-25.3)	23.4 (21.5-25.4)	<0.01
History of diabetes mellitus, %	6.6	6.5	6.4	7.6	<0.01
Family history of pancreatic cancer, %	0.3	0.4	0.4	0.3	0.84
Current smoker, %	40.2	25.9	18.8	14.6	<0.01
Ethanol intake ≥150 g/wk, %	42.4	26.1	17.6	11.6	<0.01
Coffee intake >1 cup/wk, %	76.1	76.4	73.8	67.3	<0.01
Physical activity, MET-h/d, median (IQR)	31.3 (24.1-33.7)	31.3 (24.3-33.7)	31.3 (24.7-33.1)	31.3 (24.7-33.1)	0.02
Dietary intake, median (IQR)					
Total energy (kcal/d)	1963 (1560-2458)	1954 (1565-2435)	1928 (1571-2359)	1803 (1467-2216)	<0.01
Processed meat (g/d)	3.3 (1.0-8.0)	4.3 (1.5-9.6)	4.3 (1.5-9.5)	3.3 (1.0-7.5)	<0.01
Fish (g/d)	64.9 (39.3-104.6)	80.4 (50.2-124.0)	84.1 (52.3-130.0)	75.4 (46.3-117.6)	<0.01
**α carotene (μg/day)**	< 61.5	61.5-239.1	239.1-873.0	≥ 873.0	
Age (years), median (IQR)	50 (44-57)	51 (45-57)	51 (45-57)	53 (46-58)	<0.01
Men, %	59.6	48.8	41.5	37.7	<0.01
BMI (kg/m^2), median (IQR)	23.2 (21.4-25.3)	23.3 (21.4-25.3)	23.4 (21.5-25.4)	23.4 (21.6-25.5)	<0.01
History of diabetes mellitus, %	6.9	6.3	6.4	7.6	<0.01
Family history of pancreatic cancer, %	0.3	0.4	0.4	0.3	0.78
Current smoker, %	35.9	26.2	21.2	16.3	<0.01
Ethanol intake ≥150 g/wk, %	36.2	26.2	20.7	14.6	<0.01
Coffee intake >1 cup/wk, %	74.3	76.3	74.7	68.2	<0.01
Physical activity, MET-h/d, median (IQR)	31.3 (24.1-33.7)	31.3 (24.3-33.7)	31.3 (24.7-33.7)	31.3 (24.7-33.1)	<0.01
Dietary intake, median (IQR)					
Total energy (kcal/d)	1926 (1501-2471)	1939 (1602-2387)	1992 (1609-2438)	1792 (1450-2173)	<0.01
Processed meat (g/d)	3.0 (1.0-7.4)	4.3 (1.5-9.1)	4.3 (1.7-9.6)	3.5 (1.3-8.4)	<0.01
Fish (g/d)	67.2 (40.0-110.4)	79.4 (51.3-120.8)	85.5 (52.7-132.9)	71.8 (43.7-112.7)	<0.01
**β cryptoxanthin (μg/day)**	< 85.1	85.1-414.6	414.6-1540.6	≥ 1540.6	
Age (years), median (IQR)	50 (44-56)	51 (45-57)	52 (45-58)	53 (46-59)	<0.01
Men, %	61.6	50.3	41.9	33.8	<0.01
BMI (kg/m^2), median (IQR)	23.4 (21.5-25.4)	23.3 (21.5-25.4)	23.3 (21.5-25.3)	23.3 (21.5-25.4)	0.04
History of diabetes mellitus, %	7.7	6.7	6.3	6.4	<0.01
Family history of pancreatic cancer, %	0.4	0.4	0.4	0.3	0.26
Current smoker, %	37.2	26.2	20.4	15.3	<0.01
Ethanol intake ≥150 g/wk, %	39.8	27.7	12.3	10.9	<0.01
Coffee intake >1 cup/wk, %	72.2	76.1	74.7	70.6	<0.01
Physical activity, MET-h/d, median (IQR)	31.3 (24.3-33.7)	31.3 (24.7-33.7)	31.3 (24.7-33.3)	31.3 (24.3-33.1)	<0.01
Dietary intake, median (IQR)					
Total energy (kcal/d)	1933 (1530-2437)	1991 (1610-2494)	1934 (1584-2315)	1783 (1455-2222)	<0.01
Processed meat (g/d)	3.8 (1.3-9.6)	4.3 (2.0-10.3)	4.0 (1.5-8.8)	3.0 (1.0-6.7)	<0.01
Fish (g/d)	66.3 (39.2-109.7)	80.5 (49.7-126.7)	82.4 (52.3-125.0)	74.4 (46.3-114.9)	<0.01

Baseline characteristics of participants by intake of antioxidant vitamins (continued)

	Lowest	Second	Third	Highest	P for difference
**Lycopene (μg/day)**	< 51.7	51.7-329.6	329.6-3019.5	≥ 3019.5	
Age (years), median (IQR)	52 (45-58)	51 (44-57)	51 (45-58)	51 (45-57)	<0.01
Men, %	53.0	41.9	41.5	51.2	<0.01
BMI (kg/m^2), median (IQR)	23.4 (21.5-25.5)	23.2 (21.4-25.3)	23.2 (21.4-25.2)	23.4 (21.6-25.4)	<0.01
History of diabetes mellitus, %	6.3	6.0	6.7	8.3	<0.01
Family history of pancreatic cancer, %	0.3	0.4	0.4	0.3	0.03
Current smoker, %	31.0	22.2	20.8	25.6	<0.01
Ethanol intake ≥150 g/wk, %	31.8	20.5	21.2	24.2	<0.01
Coffee intake >1 cup/wk, %	67.7	74.4	74.2	75.3	<0.01
Physical activity, MET-h/d, median (IQR)	31.3 (24.3-33.7)	31.3 (24.3-33.1)	31.3 (24.7-33.7)	31.3 (24.3-33.7)	<0.01
Dietary intake, median (IQR)					
Total energy (kcal/d)	1935 (1540-2434)	1845 (1500-2267)	2014 (1558-2610)	1876 (1560-2212)	<0.01
Processed meat (g/d)	3.3 (1.0-8.8)	3.9 (1.5-8.8)	4.1 (1.5-9.3)	3.5 (1.5-7.8)	<0.01
Fish (g/d)	68.6 (39.0-114.0)	75.3 (47.1-117.7)	85 (52.9-131.8)	74.9 (48.0-113.5)	<0.01
**α tocoqherol (mg/day)**	< 3.8	3.8-5.5	5.5-8.1	≥ 8.1	
Age (years), median (IQR)	50 (44-57)	50 (44-57)	50 (44-57)	53 (47-59)	<0.01
Men, %	61.5	44.2	40.0	42.0	<0.01
BMI (kg/m^2), median (IQR)	23.3 (21.4-25.3)	23.3 (21.5-25.3)	23.3 (21.5-25.3)	23.4 (21.5-25.5)	<0.01
History of diabetes mellitus, %	6.1	6.1	6.4	8.6	<0.01
Family history of pancreatic cancer, %	0.4	0.4	0.3	0.3	0.81
Current smoker, %	37.4	23.8	20.2	18.0	<0.01
Ethanol intake ≥150 g/wk, %	44.1	23.9	17.2	12.5	<0.01
Coffee intake >1 cup/wk, %	72.9	75.1	74.6	71.1	<0.01
Physical activity, MET-h/d, median (IQR)	31.3 (24.7-33.7)	31.3 (24.3-33.3)	31.3 (24.7-33.1)	31.3 (24.7-33.1)	<0.01
Dietary intake, median (IQR)					
Total energy (kcal/d)	1963 (1555-2422)	1887 (1533-2337)	1907 (1558-2350)	1880 (1504-2350)	<0.01
Processed meat (g/d)	3.0 (1.0-6.4)	3.8 (1.5-8.8)	4.3 (1.8-9.8)	4.3 (1.5-9.6)	<0.01
Fish (g/d)	54.2 (33-84.1)	74.3 (47.7-111.1)	87.4 (55.3-132.6)	95.8 (57.8-150.5)	<0.01
**Vitamin C (mg/day)**	< 43.3	43.3-82.7	82.7-167.5	≥ 167.5	
Age (years), median (IQR)	49 (43-55)	51 (44-57)	52 (46-58)	54 (47-59)	<0.01
Men, %	65.3	49.3	40.2	32.8	<0.01
BMI (kg/m^2), median (IQR)	23.4 (21.5-25.5)	23.3 (21.5-25.3)	23.3 (21.4-25.3)	23.3 (21.5-25.3)	<0.01
History of diabetes mellitus, %	6.7	6.7	6.7	7.1	0.15
Family history of pancreatic cancer, %	0.3	0.3	0.4	0.4	0.63
Current smoker, %	39.1	25.5	19.9	14.6	<0.01
Ethanol intake ≥150 g/wk, %	43.6	26.4	17.7	9.9	<0.01
Coffee intake >1 cup/wk, %	76	76	73.9	67.7	<0.01
Physical activity, MET-h/d, median (IQR)	31.3 (24.3-33.7)	31.3 (24.3-33.7)	31.3 (24.7-33.1)	31.3 (24.7-33.1)	0.55
Dietary intake, median (IQR)					
Total energy (kcal/d)	1969 (1565-2464)	1933 (1555-2401)	1904 (1549-2345)	1838 (1489-2265)	<0.01
Processed meat (g/d)	3.3 (1.0-8.4)	4.3 (1.5-9.6)	4.2 (1.5-9.4)	3.3 (1.0-7.4)	<0.01
Fish (g/d)	64.3 (38.7-105.0)	78.3 (48.4-122.7)	82.5 (51.9-127.3)	78.7 (49.0-120.6)	<0.01

[1] P value for difference using Kruskal-Wallis test for continuous valiables and chi-square test for categorical variables.

Supplementary Table. 2 Hazard ratio (HR) and 95% confidence interval (CI) of pancreatic cancer according to quintile of dietary vitamin intakes by smoking status

	Non-smokers			n=55,663		Current and past smokers			n=28,753	
	Q1	Q2	Q3	Q4	p trend	Q1	Q2	Q3	Q4	p trend
**Retinol Eq**										
Median intake (IQR), μg/day	379.1 (302.0-438.4)	606.6 (549.1-665.6)	865.3 (792.9-945.3)	1328.3 (1160.8-1644.1)		341.9 (253.4-415.9)	607.6 (550.7-666.4)	862.8 (792.7-942.6)	1349.3 (1168.4-1689.4)	
Cases	73	75	71	66		70	59	50	55	
Person Years	191,606	225,036	228,252	221,325		126,511	97,454	96,728	98,279	
Model 1 HR (95%CI)	Ref	0.88 (0.64-1.21)	0.82 (0.59-1.14)	0.78 (0.55-1.09)	0.13	Ref	1.06 (0.75-1.5)	0.91 (0.63-1.31)	0.95 (0.67-1.37)	0.64
Model 2 HR (95%CI)	Ref	0.87 (0.63-1.21)	0.83 (0.59-1.17)	0.80 (0.56-1.13)	0.20	Ref	0.99 (0.69-1.41)	0.84 (0.58-1.22)	0.92 (0.63-1.33)	0.49
**β carotene Eq**										
Median intake (IQR), μg/day	1741.4 (1309.6-2056.5)	3018.1 (2686.4-3346.4)	4430.7 (4037.1-4882.2)	7050.8 (6090.2-8815.4)		1554.9 (1096.9-1955.6)	2949.6 (2638-3283.6)	4383.4 (3990.6-4835.6)	6869.9 (5999.9-8611)	
Cases	55	74	73	83		76	65	47	46	
Person Years	157,875	212,484	241,144	254,714		157,354	110,754	84,231	66,633	
Model 1 HR (95%CI)	Ref	0.98 (0.69-1.40)	0.82 (0.58-1.18)	0.84 (0.59-1.19)	0.21	Ref	1.13 (0.81-1.58)	1.01 (0.70-1.46)	1.19 (0.82-1.74)	0.49
Model 2 HR (95%CI)	Ref	0.95 (0.67-1.37)	0.76 (0.53-1.10)	0.80 (0.56-1.14)	0.12	Ref	1.14 (0.81-1.61)	1.00 (0.68-1.47)	1.22 (0.82-1.80)	0.47
**α carotene**										
Median intake (IQR), μg/day	137.8 (82.7-189.3)	354.9 (298.9-416.7)	654.0 (562.9-754.2)	1278.3 (1037.4-1749.4)		119.3 (71.4-175.3)	342.2 (288.5-406.7)	638.5 (554.2-739.0)	1231.7 (1016.6-1681.8)	
Cases	60	69	75	81		69	68	50	47	
Person Years	171,199	212,500	233,351	249,167		140,986	111,570	92,551	73,864	
Model 1 HR (95%CI)	Ref	0.97 (0.69-1.37)	0.95 (0.67-1.33)	0.93 (0.66-1.31)	0.68	Ref	1.23 (0.88-1.71)	1.06 (0.74-1.53)	1.19 (0.81-1.74)	0.50
Model 2 HR (95%CI)	Ref	0.97 (0.68-1.38)	0.92 (0.64-1.30)	0.91 (0.64-1.29)	0.53	Ref	1.22 (0.86-1.73)	1.08 (0.74-1.57)	1.22 (0.82-1.81)	0.44
**β cryptoxanthin**										
Median intake (IQR), μg/day	217.4 (120.0-318.7)	630.9 (523.8-739.0)	1140.0 (988.7-1321.5)	2416.6 (1876.8-3444.0)		174.9 (89.1-295.5)	611.5 (509.5-725.0)	1114.1 (972.7-1290.4)	2311.7 (1825.2-3282.4)	
Cases	61	71	71	82		83	66	46	39	
Person Years	171842	216091	235502	242782		149561	112805	89050	67556	
Model 1 HR (95%CI)	Ref	0.90 (0.64-1.27)	0.78 (0.55-1.10)	0.82 (0.58-1.16)	0.20	Ref	0.99 (0.72-1.37)	0.83 (0.58-1.20)	0.88 (0.59-1.30)	0.34
Model 2 HR (95%CI)	Ref	0.89 (0.62-1.26)	0.79 (0.55-1.12)	0.80 (0.56-1.15)	0.20	Ref	0.95 (0.68-1.33)	0.81 (0.55-1.18)	0.89 (0.59-1.33)	0.37
**Lycopene**										
Median intake (IQR), μg/day	166.4 (81.0-246.4)	557.0 (435.3-706.1)	1553.2 (1163.0-2160.0)	5477.1 (4043.8-8126.0)		148.5 (66.1-231)	533.2 (423.6-681)	1665.9 (1201.8-2322.1)	5487.7 (4055-8108.9)	
Cases	66	71	84	64		63	48	56	67	
Person Years	195,447	227,950	233,967	208,853		122,599	94,781	91,064	110,528	
Model 1 HR (95%CI)	Ref	0.97 (0.69-1.36)	1.09 (0.79-1.51)	0.94 (0.67-1.34)	0.96	Ref	0.99 (0.68-1.44)	1.14 (0.79-1.64)	1.18 (0.84-1.68)	0.26
Model 2 HR (95%CI)	Ref	0.99 (0.70-1.40)	1.13 (0.81-1.58)	0.96 (0.67-1.37)	0.96	Ref	0.94 (0.64-1.39)	1.14 (0.79-1.64)	1.13 (0.79-1.61)	0.37
**α tocopherol**										
Median intake (IQR), mg/day	4.8 (4.2-5.2)	6.1 (5.8-6.4)	7.4 (7.0-7.7)	9.2 (8.6-10.4)		4.5 (3.8-5)	6.1 (5.8-6.4)	7.3 (7-7.7)	9.2 (8.6-10.4)	
Cases	54	74	80	77		75	56	61	42	
Person Years	171,420	222,029	235,256	237,513		148,217	102,368	88,523	79,863	
Model 1 HR (95%CI)	Ref	1.07 (0.75-1.53)	1.08 (0.76-1.53)	0.99 (0.69-1.40)	0.89	Ref	1.04 (0.73-1.47)	1.25 (0.89-1.75)	0.89 (0.61-1.31)	0.98
Model 2 HR (95%CI)	Ref	1.13 (0.78-1.64)	1.12 (0.77-1.64)	1.00 (0.68-1.49)	0.89	Ref	1.05 (0.73-1.52)	1.31 (0.90-1.90)	0.86 (0.55-1.34)	0.89
**Vitamin C**										
Median intake (IQR), mg/day	64.1 (51.0-74.1)	101.9 (92.6-110.9)	141.2 (130.6-153.4)	209.1 (185.5-246.7)		59.2 (44.5-71)	99.8 (90.9-109.5)	139.6 (129.4-151.5)	205.8 (183.8-241.5)	
Cases	45	80	74	86		71	68	48	47	
Person Years	165,312	213,428	236,634	250,843		154,485	110,745	87,863	65,879	
Model 1 HR (95%CI)	Ref	1.29 (0.89-1.87)	1.02 (0.7-1.49)	1.05 (0.72-1.53)	0.67	Ref	1.19 (0.85-1.67)	0.97 (0.67-1.41)	1.15 (0.79-1.70)	0.70
Model 2 HR (95%CI)	Ref	1.41 (0.96-2.07)	1.03 (0.69-1.54)	1.07 (0.72-1.58)	0.55	Ref	1.17 (0.83-1.65)	0.95 (0.64-1.40)	1.15 (0.77-1.72)	0.75

Model 1 was stratified by sex and study area and adjusted for age.

Model 2 was further adjusted for BMI (<25, ≥25), history of diabetes mellitus ("yes" or "no"), family history of pancreatic cancer ("yes" or "no"), ethanol intake (unknown, 0, <150g/wk, ≥150g/wk), coffee intake (<1 cup/week, ≥1 cup/week), METs, processed meat intake, and fish intake.

Supplementary Table.3 Hazard ratio (HR) and 95% confidence interval (CI) of pancreatic cancer according to quintile of dietary vitamin intakes by ethanol intake status

	< 150g/week			n=72,475		≥ 150g/week			n=22,985	
	Q1	Q2	Q3	Q4	p trend	Q1	Q2	Q3	Q4	p trend
**Retinol Eq**										
Median intake (IQR), μg/day	377.3 (296.9-437.0)	605.9 (549.1-665.6)	865.3 (793.7-945.9)	1340.6 (1166.0-1668.5)		333.3 (245.7-410.3)	608.9 (550.2-665.4)	862.8 (791.0-940.1)	1326.3 (1156.2-1656.3)	
Cases	105	100	104	106		51	46	32	31	
Person Years	227806	260575	267322	275154		109867	79368	74102	62850	
Model 1 HR (95%CI)	Ref	0.84 (0.64-1.11)	0.87 (0.66-1.14)	0.84 (0.64-1.10)	0.27	Ref	1.24 (0.83-1.85)	0.91 (0.58-1.42)	1.01 (0.64-1.58)	0.75
Model 2 HR (95%CI)	Ref	0.85 (0.64-1.13)	0.88 (0.67-1.17)	0.86 (0.65-1.14)	0.37	Ref	1.20 (0.79-1.82)	0.89 (0.56-1.41)	1.04 (0.65-1.65)	0.84
**β carotene Eq**										
Median intake (IQR), μg/day	1722.8 (1281.7-2052.1)	3016.3 (2687.5-3343.5)	4433.6 (4037.1-4881.9)	7054.3 (6087.6-8842.6)		1530.9 (1075.9-1930.8)	2931.4 (2621.4-3267.7)	4340.0 (3972.1-4811.3)	6720.8 (5964.2-8313.7)	
Cases	79	108	107	121		65	38	26	31	
Person Years	194169	251925	283012	301751		140528	87900	59178	38581	
Model 1 HR (95%CI)	Ref	1.06 (0.79-1.41)	0.91 (0.68-1.22)	0.93 (0.69-1.24)	0.38	Ref	0.87 (0.59-1.31)	0.83 (0.52-1.31)	1.43 (0.92-2.22)	0.34
Model 2 HR (95%CI)	Ref	1.08 (0.80-1.46)	0.89 (0.65-1.20)	0.92 (0.68-1.25)	0.33	Ref	0.92 (0.6-1.39)	0.90 (0.56-1.45)	1.65 (1.05-2.60)	0.11
**α carotene**										
Median intake (IQR), μg/day	132.8 (78.2-185.4)	355.7 (298.7-416.5)	652.1 (562.7-753)	1280.7 (1039.3-1755.4)		118.0 (70.1-177.0)	336.7 (286.0-403.1)	637.5 (554.0-738.2)	1193.5 (1000.6-1594.7)	
Cases	91	106	103	115		53	43	32	32	
Person Years	214537	252192	272252	291875		119976	87726	69793	48691	
Model 1 HR (95%CI)	Ref	1.06 (0.80-1.41)	0.96 (0.72-1.27)	0.98 (0.74-1.30)	0.72	Ref	1.09 (0.73-1.63)	1.00 (0.64-1.55)	1.32 (0.84-2.09)	0.35
Model 2 HR (95%CI)	Ref	1.08 (0.81-1.44)	0.95 (0.71-1.27)	1.01 (0.75-1.35)	0.81	Ref	1.14 (0.75-1.74)	1.14 (0.72-1.80)	1.57 (0.98-2.51)	0.09
**β cryptoxanthin**										
Median intake (IQR), μg/day	210.8 (116.4-314.0)	631.6 (525.0-741.1)	1140.4 (989.5-1320.2)	2432.9 (1884.2-3492.4)		170.2 (85.2-293.4)	605.2 (504.1-717.4)	1102.9 (966.3-1282)	2209.4 (1783.5-3078.3)	
Cases	89	101	99	126		65	43	29	23	
Person Years	204779	249872	278392	297812		132407	93554	64430	35796	
Model 1 HR (95%CI)	Ref	0.91 (0.68-1.21)	0.75 (0.56-1.00)	0.82 (0.62-1.09)	0.11	Ref	0.88 (0.60-1.30)	0.84 (0.54-1.30)	1.13 (0.69-1.84)	0.99
Model 2 HR (95%CI)	Ref	0.92 (0.68-1.23)	0.77 (0.57-1.03)	0.83 (0.62-1.11)	0.14	Ref	0.93 (0.62-1.38)	0.91 (0.58-1.44)	1.30 (0.79-2.14)	0.55
**Lycopene**										
Median intake (IQR), μg/day	164.0 (76.9-245.2)	555.3 (434.0-705.1)	1538.6 (1158.4-2146.9)	5704.7 (4138.1-8570.3)		142.0 (64.2-226.4)	525.8 (418.1-669.7)	1793.4 (1267.5-2406.7)	4962.0 (3864.0-7015.7)	
Cases	95	99	111	110		51	29	39	41	
Person Years	234005	271295	269598	255959		105696	68067	71909	80515	
Model 1 HR (95%CI)	Ref	0.95 (0.72-1.26)	1.04 (0.79-1.36)	1.08 (0.82-1.43)	0.47	Ref	0.90 (0.57-1.42)	1.13 (0.74-1.71)	1.11 (0.73-1.68)	0.48
Model 2 HR (95%CI)	Ref	0.95 (0.71-1.28)	1.07 (0.81-1.42)	1.10 (0.83-1.46)	0.37	Ref	0.95 (0.59-1.53)	1.24 (0.81-1.91)	1.12 (0.72-1.72)	0.42
**α tocopherol**										
Median intake (IQR), mg/day	4.8 (4.2-5.2)	6.1 (5.8-6.4)	7.4 (7.0-7.7)	9.3 (8.6-10.4)		4.4 (3.6-5.0)	6.1 (5.8-6.4)	7.3 (7.0-7.7)	9.1 (8.5-10.1)	
Cases	71	107	127	110		69	34	28	29	
Person Years	191909	261306	282572	295069		146989	80115	57845	41238	
Model 1 HR (95%CI)	Ref	1.15 (0.85-1.55)	1.24 (0.92-1.65)	0.98 (0.72-1.32)	0.83	Ref	0.86 (0.57-1.29)	0.93 (0.60-1.45)	1.23 (0.79-1.91)	0.53
Model 2 HR (95%CI)	Ref	1.26 (0.92-1.73)	1.38 (1.01-1.90)	1.04 (0.74-1.46)	0.95	Ref	0.91 (0.59-1.41)	1.01 (0.63-1.63)	1.27 (0.77-2.08)	0.41
**Vitamin C**										
Median intake (IQR), mg/day	64.4 (51.0-74.3)	101.9 (92.5-110.9)	141.2 (130.6-153.3)	209.7 (185.7-247.9)		57.8 (43.0-69.7)	99.5 (90.8-108.8)	139.0 (129.1-150.9)	200.2 (181.0-230.9)	
Cases	66	107	116	126		59	53	19	29	
Person Years	192079	251910	281676	305191		144465	88863	59942	32916	
Model 1 HR (95%CI)	Ref	1.26 (0.92-1.73)	1.09 (0.79-1.51)	0.99 (0.72-1.37)	0.49	Ref	1.31 (0.90-1.90)	0.65 (0.39-1.10)	1.70 (1.07-2.69)	0.33
Model 2 HR (95%CI)	Ref	1.26 (0.92-1.74)	1.10 (0.80-1.51)	0.99 (0.72-1.37)	0.48	Ref	1.35 (0.91-1.99)	0.69 (0.40-1.18)	1.94 (1.21-3.10)	0.14

Model 1 was stratified by sex and study area and adjusted for age.

Model 2 was further adjusted for BMI (<25, ≥25) ,smoking status (Never, Ever, Current: <20, 20-40, ≥40), history of diabetes mellitus (“yes” or “no”), family history of pancreatic cancer (“yes” or “no”), coffee intake (<1 cup/week, ≥1 cup/week), METs, processed meat intake, and fish intake.
